# Association of life's essential 8 and genetic predisposition with the risk of osteoarthritis: a prospective cohort study

**DOI:** 10.3389/fnut.2025.1642749

**Published:** 2025-09-05

**Authors:** Changyu Du, Tingting Zhou, Xiang Ji, Xiaofu Tang, Honghao Yang, Zheng Ma, Mingjie Kuang, Liangkai Chen, Xiaofeng Li, Hongfei Wang, Chengrui Yang, Xiaoming Li, Jianyong Zhao, Yang Xia

**Affiliations:** ^1^Department of Orthopedics, Hebei Province Cangzhou Hospital of Integrated Traditional Chinese Medicine-Western Medicine, Cangzhou, China; ^2^Hebei Key Laboratory of Integrated Traditional and Western Medicine in Osteoarthrosis Research, Cangzhou, China; ^3^Department of Clinical Epidemiology, Shengjing Hospital of China Medical University, Shenyang, China; ^4^Liaoning Key Laboratory of Precision Medical Research on Major Chronic Disease, Shenyang, China; ^5^Department of Orthopedics, Shandong Provincial Hospital Affiliated to Shandong First Medical University, Jinan, China; ^6^Department of Nutrition and Food Hygiene, Hubei Key Laboratory of Food Nutrition and Safety, School of Public Health, Tongji Medical College, Huazhong University of Science and Technology, Wuhan, China; ^7^Department of Hand and Microsurgery, Hebei Province Cangzhou Hospital of Integrated Traditional Chinese Medicine-Western Medicine, Cangzhou, China

**Keywords:** osteoarthritis, cardiovascular health (CVH), life's essential 8 (LE8), cohort study, genetic risk

## Abstract

**Background:**

Osteoarthritis (OA) is a prevalent joint disorder with significant health and economic impacts. While individual cardiovascular health (CVH) factors have been linked to OA, a comprehensive understanding of how overall cardiovascular health influences OA risk is lacking. Life's Essential 8 (LE8) offers a holistic measure incorporating physical activity, body mass index, diet, sleep, blood pressure, blood sugar, lipids, and nicotine exposure. The inclusion of LE8 in OA research is crucial to better understand the broader cardiovascular health-OA association.

**Methods:**

The present study was conducted using data from the UK Biobank, which initially included more than 500,000 participants. After applying exclusion criteria, a total of 242,278 participants without hip and/or knee OA at baseline were included in the final analyses. Genetic risk scores (GRSs) for hip, knee, and hip/knee OA were derived from 70, 83, and 87 single-nucleotide polymorphisms, respectively. Restricted cubic splines (RCS) were utilized to explore the potential non-linear associations of GRSs with the risks of OA. Cox proportional hazards models were applied to assess the associations between LE8, GRSs, and the risk of OA. Both combined effect and interaction analyses were conducted to evaluate how LE8 and GRSs jointly influence OA risk.

**Results:**

During a median follow-up period of 12.12 years, 18,767 participants developed hip and/or knee OA. LE8 was found to be negatively associated with the risks of hip, knee, and hip/knee OA. CVH, as measured by LE8 scores, was categorized into three groups based on the following boundary values: high CVH (80–100 points), moderate CVH (50–79 points), and low CVH (0–49 points). Compared to the participants with low CVH, the HRs (95% CIs) of hip, knee, and hip/knee OA for those with high CVH were 0.71 (0.64, 0.79), 0.48 (0.44, 0.52), and 0.56 (0.52, 0.60), respectively. These associations were not modified by GRSs. In the joint association analyses, the lowest HR (hip OA: 0.48, 0.41–0.55; knee OA: 0.34, 0.30–0.39; hip/knee OA: 0.43, 0.39–0.47) of events were observed in those with both high CVH and low GRSs, compared to those low CVH and high GRSs.

**Conclusion:**

Our findings underscore the importance of maintaining the maximum CVH to prevent the onset of OA, irrespective of genetic predisposition.

## Introduction

Osteoarthritis (OA) represents the most prevalent type of arthritis, with a particular impact on the knee and hip joints (1). This condition is the most frequently occurring joint disorder, marked by the gradual deterioration of cartilage. This progression can ultimately result in degeneration, fibrosis, fractures, and extensive harm to the joint surface (2). According to the World Health Organization, over 500 million people globally suffer from OA, with the UK having one of the highest age-standardized prevalence rates (3). Projections suggest that by 2030, the economic burden of OA in the UK could reach £3.43 billion annually (4).

A well-established relationship exists between cardiovascular disease (CVD) and OA, with OA linked to a heightened risk of early mortality due to CVD (5). Factors that contribute to CVD, such as body mass index (BMI), physical activity, and lipid levels, are also implicated in the development and progression of OA. A higher BMI, for instance, not only increases the risk of developing OA but also accelerates its progression (3). Similarly, excessive physical activity can exacerbate wear and tear on the cartilage of the knee and hip joints (6), while high blood lipid levels, often seen in obese individuals, are another risk factor for OA (7). Although earlier research has primarily concentrated on specific cardiovascular risk factors in connection with OA, the broader relationship between overall cardiovascular health and OA has not been thoroughly investigated. In 2022, the American Heart Association unveiled a thorough approach for evaluating cardiovascular health (CVH), referred to as Life's Essential 8 (LE8) (8). This approach assesses eight essential health indicators: BMI, degree of physical activity, eating patterns, sleep quality, exposure to nicotine, blood pressure, lipid profiles, and blood glucose levels (9). By examining the association between LE8 scores and OA, we can gain insights into the potential connection between cardiovascular health and OA. To the best of our understanding, several cross-sectional studies examined the impact of LE8 on OA prevalence (10–12). The results of these studies showed a negative association between the prevalence of OA and LE8 scores. Additionally, a study has focused on the association between LE8 and mortality in OA patients. This study found that high CVH was associated with a lower risk of mortality in OA patients (13). Nevertheless, because of the characteristics inherent in a cross-sectional design, there is a possibility that reversed causality may be present.

Furthermore, OA is influenced by both genetic and environmental factors. Earlier research has pinpointed specific single nucleotide polymorphisms (SNPs) that could play a role in increasing the risk of OA (14). However, genetic risk scores (GRS), which consider multiple SNPs, provide a more comprehensive assessment of overall disease risk (15). To date, no research has investigated whether the association between LE8 and the risk of OA varies among individuals with differing levels of genetic predisposition.

As a result, this research utilizes a prospective cohort design, drawing on data from the UK Biobank to investigate the connection between LE8 and the risk of OA, while also assessing whether genetic risk factors impact this association.

## Materials and methods

### Study population

The participants in this study were selected from the UK Biobank, a longitudinal cohort study which includes more than 500,000 individuals who visited 22 assessment centers across the United Kingdom between 2006 and 2010. This research offers comprehensive data for examining both genetic and environmental factors influencing a range of diseases (16). We excluded participants with missing LE8 measurements (*n* = 221,427), missing genetic information (*n* = 2,039), gender mismatch (*n* = 174), missing covariates (*n* = 2,712), other types of arthritis (*n* = 33,321), and those lost to follow-up (*n* = 666). The final sample for the cohort analysis consisted of 242,278 participants (Supplementary Figure 1). The study was granted approval by the Northwest Multi-Center Research Ethics Committee, and all participants provided informed consent at the time of their initial recruitment for the UK Biobank.

### CVH assessment with LE8

The LE8 score consists of eight distinct elements: exposure to nicotine, quality of sleep, blood lipid levels, blood glucose levels, degree of physical activity, BMI, eating patterns and blood pressure. Each component is evaluated on a scale that ranges from 0 to 100. The overall composite LE8 score, which similarly spans from 0 to 100, is calculated as the unweighted average of the scores across the eight components (17). Following the recommendations established by the American Heart Association, LE8 scores are divided into three categories: high CVH (80–100 points), moderate CVH (50–79 points), and low CVH (0–49 points) (8). Comprehensive evaluations of each component can be found in Supplementary Table 1. The dietary component of LE8 includes indicators such as whole food intake, sodium intake, fruit and vegetable consumption, and fish consumption (8). The detailed explanation of the diet score can be found in the Supplementary Table 2.

### Definition of incident OA and OA genetic risk

Based on inpatient records and death registrations from national hospitals, this study analyzed verified cases of hip and knee OA, specifically using the International Classification of Diseases, 10th edition (ICD-10) codes M16–17. In the analyses of subgroups, hip OA (ICD-10: M16) and knee OA (ICD-10: M17) were assessed separately. Genotyping and DNA array information for research in the UK Biobank has been detailed in previous publications (18). Based on findings from a prior study, a total of 83, 70, and 87 SNPs associated with knee joint, hip joint, and combined hip/knee joint OA were analyzed to assess genetic susceptibility for these three types of OA (19). Information regarding the selected SNPs can be found in Supplementary Table 3. The generation and standardized evaluation of OA-GRS is based on past effective methodological research (20). Each SNP was weighted by its effect size (β coefficient) and recoded as 0, 1, or 2 based on the number of risk alleles. The GRS was calculated using the following equation:


(1) $$\begin{array}{r} \text{GRS}=({\text{β}}_{1}\;\times \;{\text{SNP}}_{1}+\ldots +{\text{β}}_{83}\times {\text{SNP}}_{83})\\ \times (83/\text{sum of the β coefficients}) \end{array}$$

Restricted cubic splines (RCS) were utilized to explore the potential non-linear associations of GRSs with the risks of OA. For subsequent analyses, the OA-GRSs were classified into high and low categories based on their distribution, where high is defined as (GRS ≥ median) and low as (GRS < median).

### Assessment of covariates

Baseline covariates included age, gender, ethnicity, educational attainment, the Townsend deprivation index (TDI), genotyping batch, alcohol consumption status, and 10 principal components of genetic composition. Comprehensive definitions for each covariate can be found in Supplementary Method.

### Statistical analysis

For continuous variables, the mean and standard deviation (SD) were presented, and for categorical variables, the percentages were expressed by category of CVH. In order to determine differences in characteristics, variance analysis and chi-square tests were used. Cox proportional hazards models were used to evaluate the association between LE8, its subscales, and individual components with OA incidence. Hazard ratios (HRs) and corresponding 95% confidence intervals (CIs) were calculated. The Schoenfeld tests confirmed that the proportional hazards assumption was met. Furthermore, the cumulative risk curve for OA across different CVH categories was constructed utilizing the Kaplan-Meier method. Model 1 represented a basic, unadjusted framework, while Model 2 incorporated adjustments for age as a continuous variable and sex categorized as male or female. Model 3 incorporated further adjustments for race, classified as White, Asian, Black, or other; the TDI, analyzed in quartiles; drinking status, categorized as current, former, or never; and educational attainment, which was divided into low, medium, or high levels. Furthermore, the analysis also accounted for genotyping batch effects and the first ten principal components of genetic variation, particularly in relation to OA-GRSs. We also analyzed the association between each individual component of the LE8 and the incidence of OA.

We conducted a comprehensive analysis to explore genetic factors that might influence the association between LE8 scores and OA risk. Interactions between LE8 scores and OA-GRSs were assessed by adding interaction terms to the final Cox model (Model 3). We evaluated the combined effect of LE8 scores and OA-GRSs on OA risk, dividing participants into six groups based on their LE8 scores and OA-GRS categories, with the reference group being low LE8 and high OA-GRS. Four knots were automatically selected using the lowest Akaike Information Criterion, with the 10th percentile of LE8 scores as the reference point. All covariates were adjusted accordingly. Besides, we analyzed the association between each individual component of the LE8 and incidence of OA outcomes after stratifying by OA-GRS.

Our main findings were subjected to several sensitivity analyses in order to assess their reliability. Firstly, OA cases diagnosed in the first year of follow-up were excluded. Secondly, OA cases diagnosed during the first 2 years of follow-up were excluded. Thirdly, participants with a cancer history at baseline were also excluded, as this could potentially influence their CVH factors. Fourth, restricting the analysis to participants with primary care data. Fifth, further adjusting for baseline history of joint injury. Sixth, further adjusting for baseline history of diabetes and CVD.

All statistical analyses were performed using SAS software, version 9.4 (SAS Institute Inc., Cary, NC, USA). *P*-values were assessed as two-sided, with a statistical significance threshold set at 0.05.

## Results

### Baseline characteristics

This prospective cohort study involved a total of 242,278 participants. During an average follow-up duration of 12.12 years, 18,767 individuals received a diagnosis of OA. The baseline characteristics categorized by OA status are presented in Table 1. Participants exhibiting high levels of CVH were generally younger, more likely to be female, better educated, and had lower BMI, reduced TDI scores, longer sleep durations, and healthier dietary habits.

**Table 1:** Baseline characteristics of participants according to LE8 score (*n* = 242,278)^a^.

**Characteristics**	**LE8 score**			***P*-value**
	**Low (0–49)**	**Moderate (50–79)**	**High (80–100)**	
Participants, *n* (%)	12,830 (5.30)	184,553 (76.2)	44,895 (18.5)	
Age (years)	55.8 (7.65)	56.2 (8.00)	52.9 (8.19)	< 0.001
**Sex**, ***n*** **(%)**				
Females	4,857 (37.9)	88,588 (48.0)	30,591 (68.1)	<0.001
Males	7,973 (62.1)	95,965 (52.0)	14,304 (31.9)	<0.001
TDI	−0.42 (3.39)	−1.52 (2.97)	−1.78 (2.80)	<0.001
**Education**, ***n*** **(%)**				
Low	6,194 (48.3)	73,799 (40.0)	14,998 (33.4)	<0.001
Medium	5,529 (43.1)	88,190 (47.8)	22,718 (50.6)	<0.001
High	1,107 (8.63)	22,564 (12.2)	7,179 (16.0)	<0.001
**Drinking status**, ***n*** **(%)**				
Current	11,673 (91.0)	171,870 (93.1)	41,754 (93.0)	<0.001
Previous	706 (5.50)	5,716 (3.10)	1,262 (2.81)	<0.001
Never	451 (3.52)	6,967 (3.78)	1,879 (4.19)	<0.001
BMI (kg/m^2^)	32.3 (5.58)	27.6 (4.31)	23.7 (2.67)	<0.001
Healthy diet score	3.15 (1.56)	4.36 (1.65)	5.45 (1.49)	<0.001
Moderate PA (min/wk.)	77.2 (273.7)	270.5 (459.3)	320.5 (447.3)	<0.001
Vigorous PA (min/wk.)	21.6 (128.1)	89.3 (199.8)	126.1 (185.8)	<0.001
Sleep duration (hours/day)	6.88 (1.67)	7.16 (1.05)	7.32 (0.77)	<0.001
SBP (mmHg)	147.3 (17.4)	139.8 (17.9)	123.1 (13.9)	<0.001
DBP (mmHg)	88.8 (10.1)	83.6 (9.71)	74.6 (8.04)	<0.001
Non-HDL (mg/dL)	185.1 (46.1)	168.7 (40.5)	138.6 (30.8)	<0.001
Glycated hemoglobin (%)	3.79 (1.16)	3.28 (0.56)	3.09 (0.34)	<0.001

BMI, body mass index; LE8, Life's Essential 8; DBP, diastolic blood pressure; HDL, high-density lipoprotein; PA, physical activity; SBP, systolic blood pressure; TDI, Townsend Deprivation Index.

a Continuous variables were presented as mean (standard deviation) and categorical variables were shown as percentage.

### Associations between LE8 and the risk of OA

**Figure 1:**
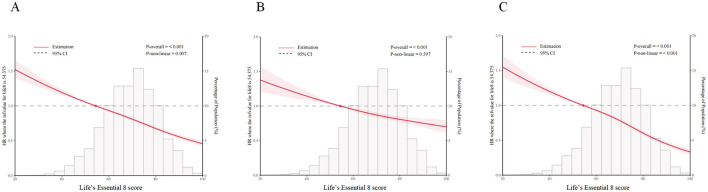
Associations of the risk of **(A)** hip and/or knee osteoarthritis, **(B)** hip osteoarthritis, and **(C)** knee osteoarthritis with Life's Essential 8 score in Cox models with restricted cubic splines. HRs, Hazard ratios; CIs, confidence intervals. The model was adjusted for age, sex, body mass index, education levels, Townsend deprivation index, ethnic background, drinking status, healthy diet score, genetic predisposition, first 10 genetic principal components, genotype measurement batch adjusted.

As illustrated in Figure 1, LE8 showed a negative association with the risks of OA in the hip, knee, and the combined hip/knee (all *P*-values for trend < 0.05). Table 2 illustrates that, in contrast to participants exhibiting low CVH, the HRs (95% CIs) of hip, knee, and hip/knee OA for those with high CVH were 0.71 (0.64, 0.79), 0.48 (0.44, 0.52), and 0.56 (0.52, 0.60), respectively. These associations were not modified by GRSs (all *P* for interactions > 0.05). The adjusted HR (95% CI) of incident hip, knee, and hip/knee OA for each SD increment in LE8 was 0.91 (0.89, 0.93), 0.80 (0.79, 0.82), 0.84 (0.83, 0.85). Furthermore, significant negative linear associations of behavior (hip OA HR: 0.90, 95% CI, 0.88–0.92 for each SD increment; knee OA HR: 0.81, 95% CI, 0.80–0.82 for each SD increment; hip/knee OA HR: 0.84, 95% CI, 0.83–0.85 for each SD increment) and biological subscales (hip OA HR: 0.97, 95% CI, 0.95–0.99 for each SD increment; knee OA HR: 0.91, 95% CI, 0.90–0.93 for each SD increment; hip/knee OA HR: 0.94, 95% CI, 0.92–0.95 for each SD increment) of LE8 and the risk of OA were observed after adjusting for potential confounders.

**Table 2:** Associations between LE8 score and the risk of OA (*n* = 242,278)^a^.

	**LE8 score**			***P* trend**	**Each SD increase in LE8 score**	**Each SD increase in behavior scale**	**Each SD increase in biological scale**
	**Low (0–49)**	**Moderate (50–79)**	**High (80–100)**				
**Participants**	12,830	184,553	44,895		242,278	242,278	242,278
**Osteoarthritis of hip and/or knee**							
Cases, *n*	1,278	15,169	2,320		18,767	18,767	18,767
Person-years	140,911	2,080,230	516,904		2,738,045	2,738,045	2,738,045
Model 1^b^	1.00 (reference)	0.80 (0.76, 0.85)	0.49 (0.46, 0.53)	<0.001	0.82 (0.81, 0.83)	0.87 (0.86, 0.89)	0.83 (0.81, 0.84)
Model 2^c^	1.00 (reference)	0.76 (0.72, 0.80)	0.55 (0.51, 0.58)	<0.001	0.84 (0.83, 0.85)	0.84 (0.83, 0.85)	0.93 (0.92, 0.95)
Model 3^d^	1.00 (reference)	0.77 (0.73, 0.81)	0.56 (0.52, 0.60)	<0.001	0.84 (0.83, 0.85)	0.84 (0.83, 0.85)	0.94 (0.92, 0.95)
**Osteoarthritis of hip**							
Cases, *n*	475	6,127	1,116		7,718	7,718	7,718
Person-years	145,681	2,135,166	524,339		2,805,186	2,805,186	2,805,186
Model 1	1.00 (reference)	0.88 (0.80, 0.96)	0.65 (0.58, 0.72)	<0.001	0.88 (0.87, 0.91)	0.95 (0.93, 0.97)	0.85 (0.83, 0.87)
Model 2	1.00 (reference)	0.81 (0.73, 0.89)	0.71 (0.63, 0.79)	<0.001	0.91 (0.89, 0.93)	0.90 (0.88, 0.92)	0.98 (0.95, 1.00)
Model 3	1.00 (reference)	0.81 (0.74, 0.89)	0.71 (0.64, 0.79)	<0.001	0.91 (0.89, 0.93)	0.90 (0.88, 0.92)	0.97 (0.95, 0.99)
**Osteoarthritis of knee**							
Cases, *n*	855	9,744	1,288		11,887	11,887	11,887
Person-years	143,256	2,110,094	522,304		2,775,654	2,775,654	2,775,654
Model 1	1.00 (reference)	0.77 (0.72, 0.83)	0.41 (0.38, 0.45)	<0.001	0.78 (0.77, 0.79)	0.83 (0.81, 0.84)	0.81 (0.80, 0.83)
Model 2	1.00 (reference)	0.75 (0.70, 0.80)	0.46 (0.42, 0.51)	<0.001	0.80 (0.78, 0.81)	0.81 (0.79, 0.82)	0.91 (0.89, 0.93)
Model 3	1.00 (reference)	0.76 (0.71, 0.81)	0.48 (0.44, 0.52)	<0.001	0.80 (0.79, 0.82)	0.81 (0.80, 0.82)	0.91 (0.90, 0.93)

a LE8, Life's Essential 8; OA, Osteoarthritis; SD, standard deviation.

b Model 1 was a crude model.

c Model 2 was adjusted for age (continuous) and sex (male or female).

d Model 3 was further adjusted for race (White, Asian, Black, or others), Townsend deprivation index (categorical, quartiles), drinking status (current, previous, or never), and education levels (low, moderate, or high).

When we analyzed the individual component of LE8, high diet score, high sleep health, high BMI score (low BMI level), high blood glucose score (low glucose level) and high blood pressure score (low blood pressure level) were associated with significant lower risks of hip/knee OA. Besides, high nicotine exposure score, high sleep health, high BMI score (low BMI level), and high blood pressure score (low blood pressure level) were associated with significant lower risks of hip OA. High diet score, high sleep health, high BMI score (low BMI level), high blood glucose score (low glucose level) and high blood pressure score (low blood pressure level) were associated with significant lower risks of knee OA (Supplementary Table 4).

### Associations between LE8 and the risk of OA according to OA-GRSs

Supplementary Figure 2 showed non-linear associations of GRS-hip with the risks of OA (*P*-values for non-linear < 0.001) and linear associations of GRS-knee and GRS-hip/knee with the risks of OA (*P*-values for non-linear >0.05). Supplementary Table 5 indicated a positive correlation between OA-GRSs and the risks associated with hip and/or knee OA. Compared to participants with low OA-GRSs, those with high OA-GRSs had a 47% (HR, 1.47; 95% CI, 1.41–1.54), 26% (HR, 1.26; 95% CI, 1.21–1.30), and 25% (HR, 1.25; 95% CI, 1.22–1.29) increased risks of hip, knee, and hip/knee OA, respectively. No significant interaction on incident OA was found among LE8 categories and OA-GRSs (Table 3). Supplementary Table 6 presents the association between each individual component of LE8 and the incidence of OA outcomes after stratifying by OA-GRS. In the low OA-GRS group, diet, physical activity, sleep, BMI, and blood pressure were associated with the incidence of OA of the hip and/or knee. Specifically, physical activity, sleep, and BMI were associated with hip OA, while diet, physical activity, nicotine exposure, sleep, BMI, blood glucose, and blood pressure were associated with knee OA. In the high OA-GRS group, sleep, BMI, and blood pressure were associated with the incidence of hip and/or knee OA. Nicotine exposure, sleep, and BMI were associated with hip OA, whereas diet, physical activity, nicotine exposure, sleep, BMI, blood glucose, and blood pressure were associated with knee OA. However, no significant interaction on incident OA were found between individual components of LE8 and OA-GRS.

**Table 3:** Associations between LE8 score and the risks of OA of hip and knee according to GRS (*n* = 242,278)^a^.

	**LE8 score**			***P* trend**	**Each SD increase in LE8 score**	**Each SD increase in behavior scale**	**Each SD increase in biological scale**
	**Low (0–49)**	**Moderate (50–79)**	**High (80–100)**				
**Osteoarthritis of hip and/or knee**							
**Low genetic risk**							
Cases/Participants, *n*	533/6,136	6,855/92,345	1,031/22,645		8,419/121,126	8,419/121,126	8,419/121,126
Person-years	67,959	1,045,097	261,517		1,374,573	1,374,573	1,374,573
Model 1^b^	1.00 (reference)	0.84 (0.76, 0.91)	0.50 (0.45, 0.57)	<0.001	0.81 (0.80, 0.83)	0.87 (0.86, 0.89)	0.82 (0.80, 0.84)
Model 2^c^	1.00 (reference)	0.79 (0.72, 0.86)	0.56 (0.50, 0.62)	<0.001	0.84 (0.82, 0.85)	0.84 (0.82, 0.86)	0.93 (0.91, 0.95)
Model 3^d^	1.00 (reference)	0.80 (0.73, 0.87)	0.57 (0.51, 0.64)	<0.001	0.84 (0.82, 0.86)	0.84 (0.83, 0.86)	0.93 (0.91, 0.95)
**High genetic risk**							
Cases/Participants, *n*	745/6,694	8,314/92,208	1,289/22,250		10,348/121,152	10,348/121,152	10,348/121,152
Person-years	72,952	1,035,133	255,387		1,363,472	1,363,472	1,363,472
Model 1	1.00 (reference)	0.79 (0.73, 0.85)	0.49 (0.45, 0.54)	<0.001	0.82 (0.81, 0.84)	0.87 (0.86, 0.89)	0.83 (0.82, 0.85)
Model 2	1.00 (reference)	0.75 (0.69, 0.80)	0.54 (0.49, 0.59)	<0.001	0.84 (0.82, 0.86)	0.84 (0.83, 0.86)	0.94 (0.92, 0.96)
Model 3	1.00 (reference)	0.75 (0.70, 0.81)	0.55 (0.50, 0.60)	<0.001	0.84 (0.83, 0.86)	0.84 (0.83, 0.86)	0.94 (0.92, 0.96)
**Osteoarthritis of hip**							
**Low genetic risk**							
Cases/Participants, *n*	180/6,261	2,503/92,396	452/22,472		3,135/121,129	3,135/121,129	3,135/131,129
Person-years	71,604	1,072,197	262,795		1,406,596	1,406,596	1,406,596
Model 1	1.00 (reference)	0.93 (0.80, 1.08)	0.68 (0.58, 0.81)	<0.001	0.89 (0.86, 0.92)	0.97 (0.93, 1.00)	0.84 (0.81, 0.87)
Model 2	1.00 (reference)	0.84 (0.72, 0.98)	0.74 (0.62, 0.88)	<0.001	0.92 (0.88, 0.95)	0.91 (0.88, 0.95)	0.97 (0.93, 1.01)
Model 3	1.00 (reference)	0.86 (0.74, 0.99)	0.75 (0.63, 0.90)	<0.001	0.92 (0.89, 0.96)	0.92 (0.89, 0.95)	0.97 (0.93, 1.01)
**High genetic risk**							
Cases/Participants, *n*	295/6,569	3,624/92,517	664/22,423		4,583/121,149	4,583/121,149	4,583/121,149
Person-years	74,077	1,062,969	261,544		1,398,590	1,398,590	1,398,590
Model 1	1.00 (reference)	0.85 (0.76, 0.96)	0.64 (0.55, 0.73)	<0.001	0.88 (0.86, 0.91)	0.95 (0.92, 0.97)	0.85 (0.83, 0.88)
Model 2	1.00 (reference)	0.79 (0.70, 0.89)	0.69 (0.60, 0.79)	<0.001	0.90 (0.88, 0.93)	0.89 (0.87, 0.92)	0.98 (0.95, 1.01)
Model 3	1.00 (reference)	0.79 (0.70, 0.89)	0.69 (0.60, 0.79)	<0.001	0.90 (0.88, 0.93)	0.89 (0.87, 0.92)	0.98 (0.95, 1.01)
**Osteoarthritis of knee**							
**Low genetic risk**							
Cases/Participants, *n*	350/6,191	4,398/92,270	542/22,687		5,290/121,148	5,290/121,148	5,290/121,148
Person-years	69,467	1,057,420	264,194		1,391,081	1,391,081	1,391,081
Model 1	1.00 (reference)	0.82 (0.74, 0.92)	0.41 (0.36, 0.47)	<0.001	0.77 (0.75, 0.79)	0.83 (0.81, 0.85)	0.80 (0.78, 0.82)
Model 2	1.00 (reference)	0.80 (0.71, 0.89)	0.46 (0.40, 0.53)	<0.001	0.79 (0.77, 0.81)	0.80 (0.78, 0.83)	0.90 (0.87, 0.92)
Model 3	1.00 (reference)	0.81 (0.72, 0.90)	0.48 (0.42, 0.55)	<0.001	0.80 (0.78, 0.82)	0.80 (0.79, 0.83)	0.90 (0.88, 0.93)
**High genetic risk**							
Cases/Participants, *n*	505/6,639	5,346/92,283	746/22,208		6,597/121,130	6,597/121,130	6,597/121,130
Person-years	73,789	1,052,674	258,110		1,384,573	1,384,573	1,384,573
Model 1	1.00 (reference)	0.74 (0.68, 0.81)	0.42 (0.38, 0.47)	<0.001	0.79 (0.77, 0.81)	0.83 (0.81, 0.85)	0.83 (0.81, 0.85)
Model 2	1.00 (reference)	0.72 (0.65, 0.78)	0.47 (0.42, 0.53)	<0.001	0.81 (0.79, 0.83)	0.81 (0.79, 0.83)	0.92 (0.89, 0.94)
Model 3	1.00 (reference)	0.72 (0.66, 0.79)	0.48 (0.43, 0.54)	<0.001	0.81 (0.79, 0.83)	0.81 (0.79, 0.83)	0.92 (0.90, 0.95)

a LE8, Life's Essential 8; OA, Osteoarthritis; GRS, Genetic risk scores; SD, standard deviation.

P for interaction was assessed by adding the multiplicative interaction terms of LE8 scores with GRSs in the models. The P values for interaction between hip/knee, hip, and knee OA-GRSs and LE8 were 0.95, 0.66, and 0.38, respectively.

b Model 1 was a crude model.

c Model 2 was adjusted for age (continuous) and sex (male or female).

d Model 3 was further adjusted for race (White, Asian, Black, or others), Townsend deprivation index (categorical, quartiles), drinking status (current, previous, or never), education levels (low, moderate, or high), genotyping batch, and the first 10 principal components of genetics.

**Figure 2:**
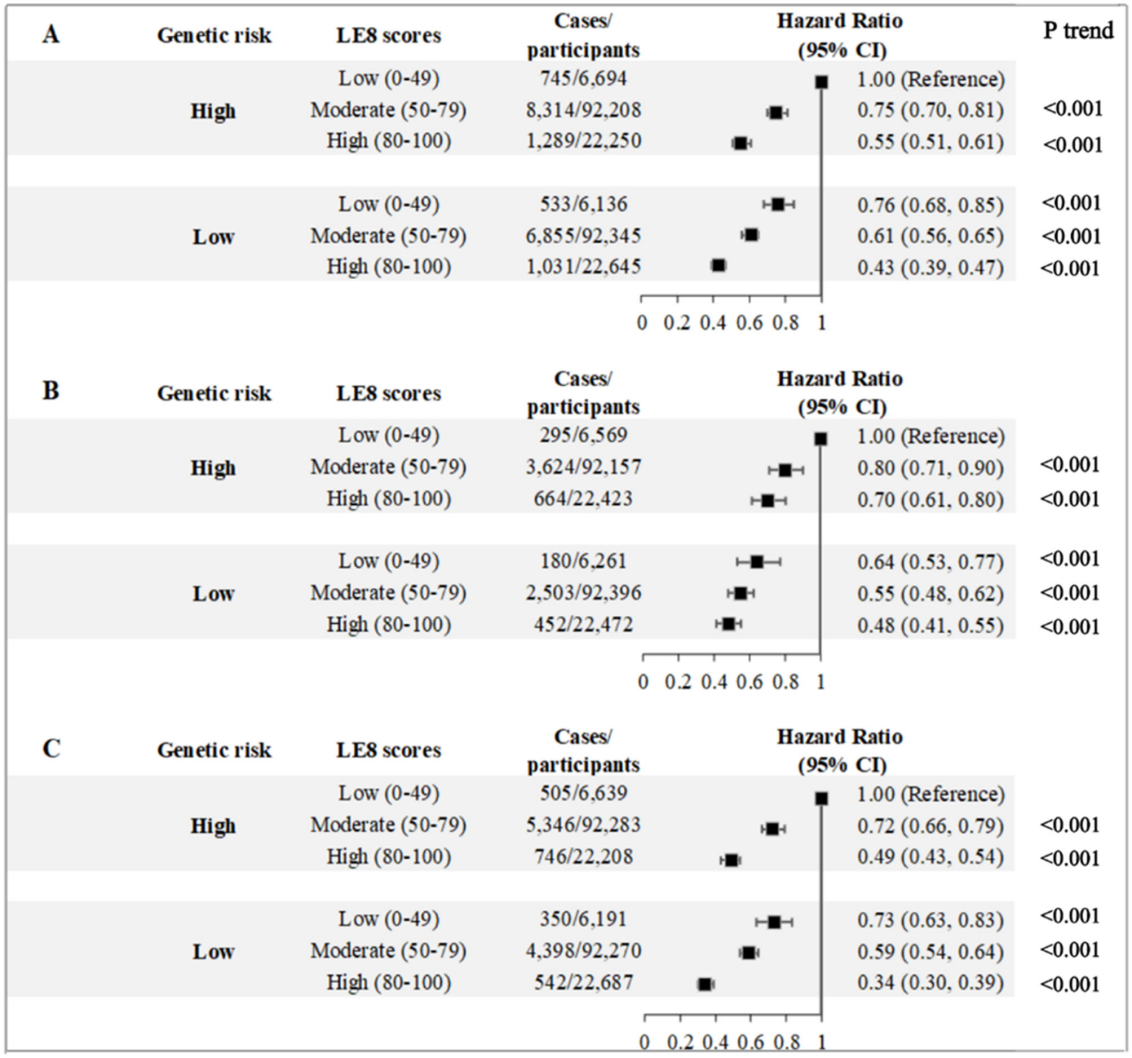
Joint associations of Life's Essential 8 score with the risks of **(A)** hip and/or knee osteoarthritis, **(B)** hip osteoarthritis, and **(C)** knee osteoarthritis. LE8, Life's Essential 8; GRS, Genetic risk scores; HRs, Hazard ratios; CIs, confidence intervals. The model was adjusted for age, sex, body mass index, education levels, Townsend deprivation index, ethnic background, drinking status, healthy diet score, genetic predisposition, first 10 genetic principal components, genotype measurement batch adjusted.

In light of the linear associations observed between LE8 categories and the risk of OA, we conducted a further investigation into the combined effects of LE8 categories and OA-GRSs on the likelihood of developing incident OA (Figure 2). As shown, the lowest HR (hip OA: 0.48, 95% CI, 0.41–0.55; knee OA: 0.34, 95% CI, 0.30–0.39; hip/knee OA: 0.43, 95% CI, 0.39–0.47) of events were noted among individuals exhibiting both high CVH and low GRSs, in contrast to those with low CVH and elevated GRSs.

### Secondary analyses

Sensitivity analyses supported the robustness of the main findings. Consistent results were observed when: (1) excluding cases of OA identified during the initial year of follow-up; (2) excluding OA cases diagnosed during the initial 2 years of the follow-up period; (3) excluding participants with a cancer history recorded at the baseline evaluation; (4) limiting the analysis to individuals who possess primary care data; (5) further adjusting for baseline history of joint injury; (6) further adjusting for baseline history of diabetes and CVD (Supplementary Table 7).

## Discussion

This comprehensive cohort study indicated that enhanced CVH, as evidenced by elevated LE8 scores, was associated with a lower risk of OA. These association were stable across various genetic risk categories for OA, showing no significant interactions. Participants displaying both low genetic risk and high CVH showed the lowest likelihood of developing OA, in contrast to those with high genetic risk and low CVH.

Several cross-sectional studies have examined the association between LE8 and OA prevalence, showing a negative association between higher LE8 scores and the prevalence of OA (10–12). These findings align with the results from our study, which supports the hypothesis that better CVH, as measured by LE8, is associated with a lower risk of OA. Moreover, a study that focused on the association between LE8 and mortality in OA patients found that higher CVH was linked to a lower risk of mortality in OA patients (13). This suggests that the protective effects of LE8 extend beyond the prevention of OA and may also influence its clinical outcomes, including mortality. However, there are several limitations in the existing literature. Most prior studies, including those based on NHANES, have relied on cross-sectional data, limiting their ability to infer causality. In contrast, our study used a large-scale prospective design with long-term follow-up, allowing us to clarify the temporal association between LE8 and incident OA. This helps address reverse causality, as individuals with preclinical OA may modify their health behaviors in ways that distort cross-sectional associations. Moreover, our study is the first to examine whether the association between LE8 and OA risk is modified by genetic susceptibility using validated polygenic risk scores. We found no significant interaction, suggesting that the protective effects of LE8 are robust across different levels of genetic risk. This finding highlights the universal value of lifestyle optimization in OA prevention, regardless of inherited predisposition.

In our study, we found that components of LE8—such as BMI, diet, blood pressure, and sleep—were independently associated with OA risk across different genetic risk levels, highlighting the importance of optimizing these factors to prevent OA. Specifically, elevated BMI and poor dietary habits were strongly associated with OA risk, likely through increased systemic inflammation and metabolic dysfunction. Furthermore, sleep quality was consistently linked to OA, with poor sleep contributing to elevated inflammatory markers and metabolic dysregulation, both of which exacerbate joint degradation.

There are several potential explanations for the results observed. As we all know, diet plays a pivotal role in determining the levels of inflammatory markers within the body. Unhealthy dietary patterns, which elevate converge on canonical inflammatory signaling, particularly the NF-κB and IL-6/IL-1β axes, are believed to activate specific inflammatory pathways that contribute to the development of OA (21–23). In parallel, IL-6 activates JAK/STAT3 signaling, which contributes to extracellular matrix breakdown, pain sensitization, and aberrant nerve ingrowth in joint tissues (24). In contrast, anti-inflammatory diets—such as the Mediterranean diet—may offer protection by reducing systemic inflammation, mitigating metabolic syndrome and obesity, and providing antioxidant benefits (25, 26). Specific nutrients have also been shown to modulate inflammatory responses. For example, foods rich in omega-3 fatty acids (e.g., salmon, flaxseeds, walnuts), polyphenols (e.g., berries, green tea, dark chocolate), and vitamins A and D exhibit anti-inflammatory properties that may counteract OA progression (22, 25, 27). Conversely, diets high in saturated fats, trans fats, and refined carbohydrates—commonly found in red meat, processed foods, and sugary beverages—are associated with increased inflammatory burden and may elevate the risk of OA (25, 28).

Besides, the impact of BMI on OA risk is multifaceted. On one hand, a high BMI leads to obesity, which increases mechanical load on weight-bearing joints such as the knee and contributes to cartilage damage, a key cause of OA (29, 30). On the other hand, obesity also fosters a state of chronic low-grade inflammation (31). Excessive adipose tissue interferes with normal metabolic signaling pathways, including insulin resistance, and promotes ectopic fat storage (32–34). The disruption of insulin signaling impairs glucose uptake and fat metabolism, which contributes to metabolic dysfunction and amplifies OA progression (28). And more, obesity, metabolic syndrome, and OA are all related to an increase in oxidative stress (35). In the presence of IL-1 or of high glucose or fatty acid levels, chondrocytes, osteoblasts, and synovial cells overproduce ROS, which, in turn, oxidize numerous proteins, lipids, and nucleic acids, thereby altering their structure and function (36, 37). This combined state of chronic inflammation and oxidative stress—particularly in individuals with high visceral fat—disrupts the normal metabolic activity of chondrocytes, weakens cartilage repair capacity, and accelerates joint degeneration (28, 29).

Interestingly, our study found that knee OA was more strongly associated with LE8 scores than hip OA. This difference may be partly explained by the knee joint's greater exposure to mechanical stress in daily life and its higher susceptibility to low-grade systemic inflammation (22). In addition, the knee appears to be more sensitive to metabolic disturbances—such as obesity, poor glycemic control, and pro-inflammatory diets—which are key components of LE8 (38). These findings suggest that adherence to a healthy lifestyle may have a more pronounced impact on knee OA prevention, highlighting the potential for targeted public health interventions aimed at modifiable lifestyle factors.

In addition to environmental factors, OA is also influenced by genetic predisposition (39). We acknowledge emerging evidence supporting potential interaction mechanisms between genetic predisposition and lifestyle factors. Several SNPs in our OA-GRS map to loci with established roles in cartilage biology and inflammation. For example, variants in IL-6/IL-1β may amplify cytokine-mediated cartilage catabolism under adverse lifestyle exposures, while favorable adherence to LE8 components could attenuate this response (19, 40). Likewise, SNPs near *MMP13* and *COL2A1* suggest that metabolic disturbances could exacerbate genetically driven matrix degradation via NF-κB–mediated pathways (41). The *GDF5* variant may modulate chondrocyte mechanosensitivity, indicating that physical activity—or lack thereof—might differentially affect cartilage integrity based on genotype (42). However, we found no significant modification of the LE8–OA association by genetic risk factors, suggesting that LE8 may benefit individuals regardless of genetic background. Nevertheless, this finding should be interpreted with caution. One plausible explanation is that the UK Biobank genotyping platform relied on genotyping array, which may lack the sequencing depth to capture rare or low-frequency variants that could interact with LE8. Future studies using whole-genome sequencing could better capture such variants and clarify gene–environment interactions.

Our findings suggest that the LE8 framework, although originally designed for cardiovascular health promotion, may also serve as a valuable tool in the primary prevention of OA. Specifically, improving individual LE8 components—such as maintaining a healthy BMI to reduce joint loading, engaging in regular physical activity to enhance joint mobility and muscle strength, optimizing blood glucose and blood pressure levels to mitigate systemic inflammation, and adopting a heart-healthy diet rich in antioxidants and anti-inflammatory nutrients—may collectively reduce the risk of OA development. These results highlight the potential clinical utility of LE8 in identifying individuals at elevated OA risk and guiding personalized, lifestyle-based preventive strategies. From a public health perspective, integrating LE8 assessment into routine health screenings could support broader, multisystem disease prevention efforts, offering a unified approach to reducing the burden of both cardiovascular disease and OA.

To the best of our understanding, this research constitutes the first examination of the connections between LE8, genetic risk factors, and the onset of OA. A large prospective cohort, a lengthy follow-up period, extensive subgroup analyses, extensive adjustments, and genetic factors all contribute to the strength of this research. Nonetheless, it is important to acknowledge several potential limitations associated with our study. First, the behavioral data on diet and sleep were self-reported, which may introduce measurement bias due to recall errors or social desirability bias. Participants may not accurately recall their dietary intake or sleep patterns, potentially leading to misclassification and attenuation of the observed associations with OA risk. Within the LE8 framework, the diet score was derived from self-reported dietary questionnaires, which is particularly susceptible to such bias. To address this limitation, future studies should consider incorporating objective measures—such as dietary and nutritional biomarkers (e.g., plasma carotenoids, omega-3 fatty acids), serum inflammatory markers (e.g., cytokine profiles), accelerometer-based physical activity monitoring, and sleep actigraphy—to validate self-reported data and provide a more robust understanding of the biological mechanisms linking cardiovascular health to OA risk (43). Second, our analysis was based on baseline LE8 measurements and did not capture temporal changes in cardiovascular health behaviors. Such dynamic changes could influence the cumulative exposure to protective or detrimental factors related to OA risk. Future longitudinal cohort studies and randomized intervention trials are warranted to examine whether improvements or deteriorations in LE8 components over time modify OA risk trajectories. Third, although we accounted for multiple confounding variables, there may still be unmeasured or residual confounding present. Fourth, the UK Biobank primarily consists of white participants and individuals with higher levels of education, which could restrict the broader applicability of our findings (44). To improve generalizability, future studies should include more racially and ethnically diverse populations, particularly those underrepresented in biomedical research, to confirm whether the observed associations persist across ancestry groups. Fifth, the study design was observational, and as such, there is a potential risk of reverse causality. Specifically, pre-existing joint pain or undiagnosed early-stage OA may have influenced LE8 components such as physical activity levels, as individuals experiencing pain might reduce their physical activity and thus receive lower LE8 scores. To mitigate this concern, we conducted sensitivity analyses excluding participants who developed OA within the first 2 years of follow-up, and the results remained consistent. Nevertheless, future longitudinal interventional studies are warranted to determine whether improving individual LE8 components can causally reduce OA incidence and whether these effects differ by joint site or genetic background. Such studies would help strengthen causal inference and inform personalized prevention strategies.

## Conclusions

Our research indicates that sustaining optimal CVH is essential for preventing OA in the overall population, irrespective of genetic background. These findings underscore the vital significance of improving LE8 scores in developing effective strategies to prevent OA by “nutrition-metabolism-OA.”

## Data Availability

Publicly available datasets were analyzed in this study. This data can be found at: data are available in a public, open access repository. This research has been conducted using the UK Biobank Resource under Application Number 63454. The UK Biobank data are available on application to the UK Biobank (www.ukbiobank.ac.uk/).
