# Supplementary material for: Association of life's essential 8 and genetic predisposition with the risk of osteoarthritis: a prospective cohort study
Source: Front Nutr. 2025 Sep 5;12:1642749. doi: 10.3389/fnut.2025.1642749 (PMC12447727; doi:10.3389/fnut.2025.1642749)
Supplement: Supplementary file 1 [file Table_1.docx]

**Supplementary Appendix**

Contents

**Supplementary Method.** Detailed definitions of covariates.

Supplementary Table 1**.** Quantitative Assessment of Life’s Essential 8 (LE8).

Supplementary Table 2**.** Healthy Diet Score using touchscreen questionnaire in the UK biobank study.

Supplementary Table 3**.** Single nucleotide polymorphisms (SNPs) used to build the GRS in the UK Biobank Study for hip and/or knee OA.

Supplementary Table 4**.** Associations Between Components of LE8 and Osteoarthritis.

Supplementary Table 5**.** Associations of GRS with the risks of the osteoarthritis.

Supplementary Table 6**.** Associations Between Components of LE8 and Osteoarthritis stratifying by GRS.

Supplementary Table 7**.** Sensitivity analyses of the association between LE8 and the risk of osteoarthritis.

**Supplementary Figure 1.** Selection of study participants.

**Supplementary Figure 2.** Associations of the risk of (A) hip and/or knee osteoarthritis, (B) hip osteoarthritis, and (C) knee osteoarthritis with GRS with restricted cubic splines.

**STROBE Statement**—checklist of items that should be included in reports of observational studies

**Supplementary Method. Detailed definitions of covariates.**

Sociodemographic characteristics included age (continuous in years), sex (men; women), BMI (calculated by dividing an individual’s weight (kg) by the square of their height (m^2^)). Education levels were categorized as “no qualifications”, “other qualifications than college/university degree”, and “college or university degree”. The Townsend Deprivation Index (TDI) was derived from the postcode of residence using aggregated data on unemployment, car and home ownership, and household overcrowding [1], with elevated scores indicating increased deprivation. Ethnicity was categorized into seven categories including “white”, “mixed”, “Asian”, “Black”, “Chinese”, “Others”, and “unknown”. The smoking and drinking status were divided into “never,” “ever,” and “current” categories. Additional covariates included walking pace (slow, steady, or fast) and the type of accommodation (house/bungalow, flat/marionette/apartment, or others). A healthy diet score was calculated based on a baseline food frequency questionnaire, as described in a previous study [2]. The total score for a healthy diet ranged from 0 to 5, with a higher score indicating a healthier diet. Finally, we adjusted for total accelerometer wear days, and gene risk scores through health records at the time of baseline assessment.

1. Health and Deprivation P Townsend P Phillimore A Beattie Health and Deprivation Published by Croom Helm 212pp £19.95 0-7099-4351-2 [Formula: see text]. *Nurs Stand* 1988, 2(17):34.

2. Wang M, Zhou T, Li X, Ma H, Liang Z, Fonseca VA, Heianza Y, Qi L: Baseline Vitamin D Status, Sleep Patterns, and the Risk of Incident Type 2 Diabetes in Data From the UK Biobank Study. *Diabetes Care* 2020, 43(11):2776-2784.


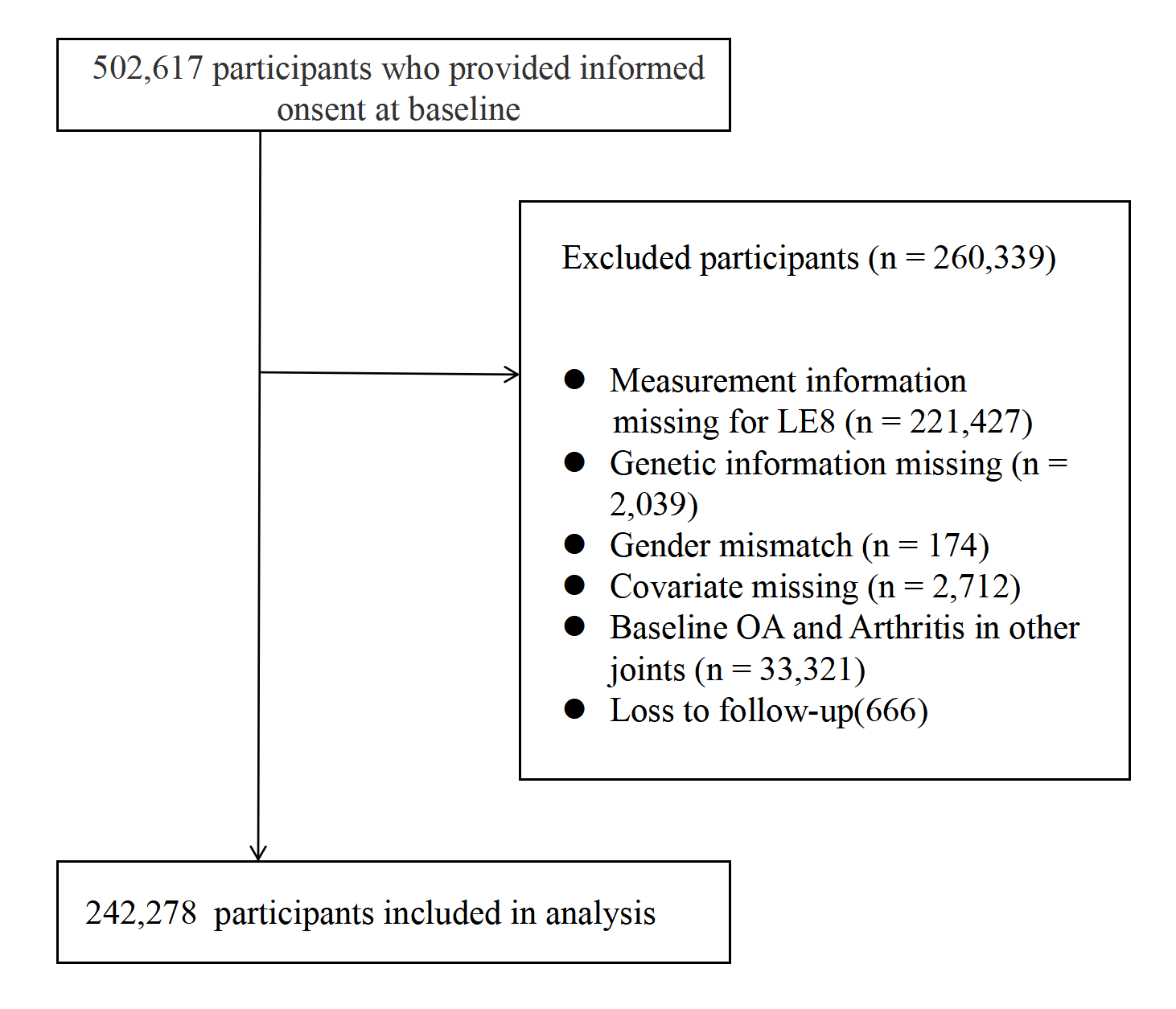


**Supplementary Figure 1.** Selection of study participants.


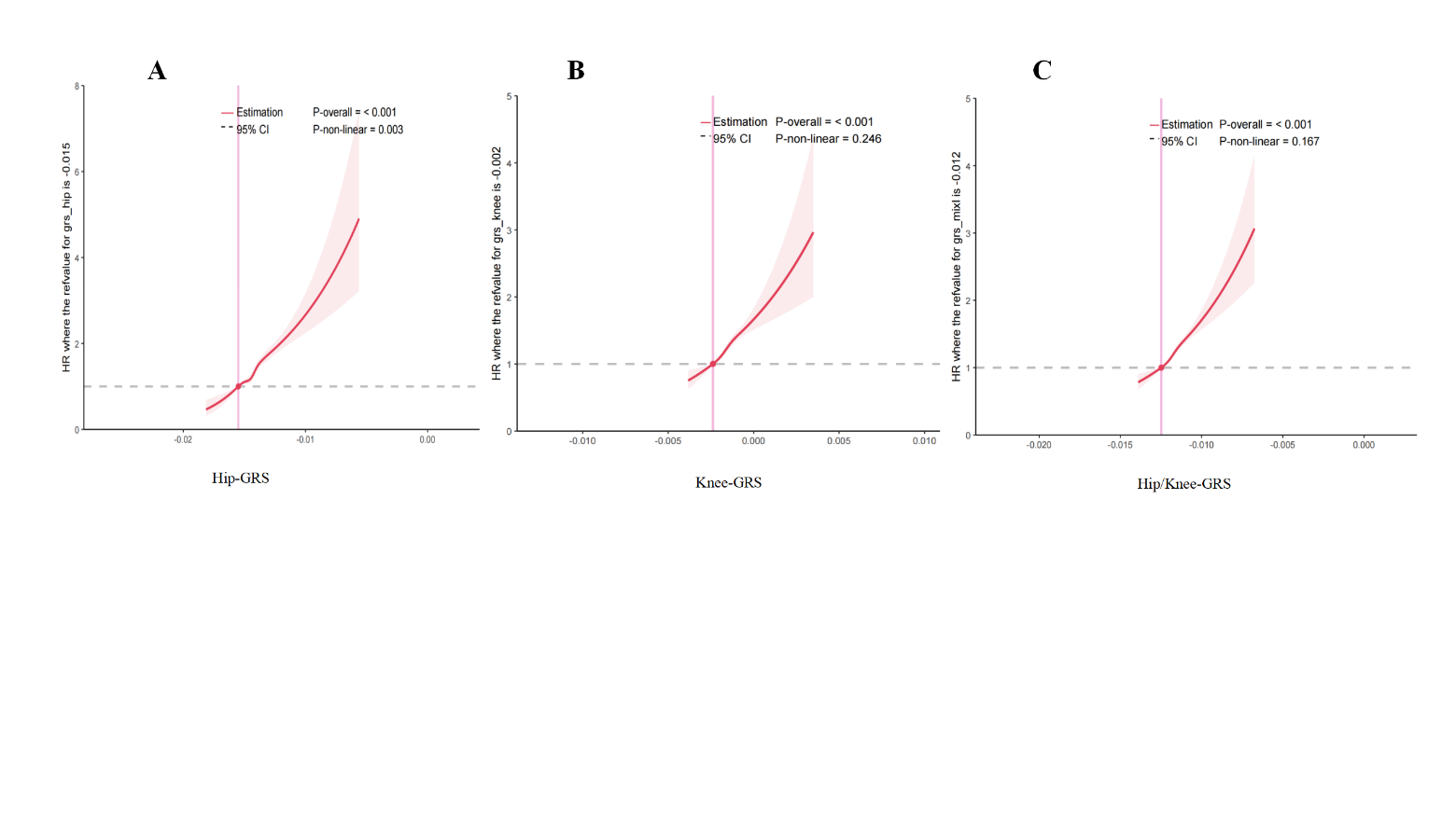


**Supplementary Figure 2.** Associations of the risk of (A) hip osteoarthritis, (B) knee osteoarthritis, and (C) hip and/or knee osteoarthritis with GRS with restricted cubic splines.

**Supplementary Table 1.** Quantitative Assessment of Life’s Essential 8 (LE8).

| LE8 metric | Method of measurement | Quantification of LE8 metric | Score and classification |
| --- | --- | --- | --- |
| Diet | Measurement: Healthy Diet Score | Scoring:  Points diets score (points)  100 8–10  80 6–7  50 4–5  25 2–3  0 0–1 | The LE8 score is scaled from 0 to 100 points, calculated as the unweighted average of all 8 component metric scores. In the present study, overall LE8 scores of 80 to 100 are considered High CVH; 50 to 79, Moderate CVH; and 0 to 49 points, low CVH. |
| Physical activity (PA) | Self-reported minutes of moderate or vigorous PA per week (1 minute of vigorous physical activity is equivalent to 2 minutes of moderate physical activity). | Scoring:  Points Minutes  100 ≥150  90 120–149  80 90–119  60 60–89  40 30–59  20 1–29  0 0 |  |
| Tobacco/nicotine exposure | Self-reported use of cigarettes; or secondhand smoke exposure (Participants were asked “Does anyone in your household smoke”. Secondhand smoke exposure was defined as if participants’ responses are “yes, one household member smokes” or “Yes, more than one household member smokes”). | Scoring:  Points Status  100 Never smoker  75 Former smoker, quit ≥5 y  50 Former smoker, quit 1–<5 y  25 Former smoker, quit <1 y  0 Current smoker  Subtract 20 points (unless score is 0) for living with active indoor smoker in home. Furthermore, only individuals who indicated they “smoked on most or all days in the past” have access to information on the precise time to quit smoking. We consider participants who indicated “smoked occasionally in the past” as equivalent to “Former smoker, quit 1–<5 years”; We consider the participants who indicated “just tried once or twice in the past” as equivalent to “Former smoker, quit ≥ 5 years”. |  |
| Sleep health | Self-reported average hours of sleep per night | Scoring:  Points Level  100 7–<9  90 9–<10  70 6–<7  40 5–<6 or ≥10  20 4–<5  0 <4 |  |
| Body mass index | Measurement: Body weight (kilograms)  divided by height squared (meters  squared) | Scoring:  Points Level  100 <25  70 25.0–29.9  30 30.0–34.9  15 35.0–39.9  0 ≥40.0 |  |
| Blood lipids (non-HDL cholesterol) | Measurement: Plasma total and HDL cholesterol with calculation of non–HDL cholesterol | Metric: Non–HDL cholesterol (mg/dL)  Scoring:  Points Level  100 <130  60 130–159  40 160–189  20 190–219  0 ≥220  If drug-treated level, subtract 20 points |  |
| Blood glucose | Measurement: HbA1c (%) and history of diabetes | Metric: HbA1c (%)  Scoring:  Points Level  100 No history of diabetes and HbA1c < 5.7  60 No diabetes and HbA1c 5.7–6.4 (prediabetes)  40 Diabetes with HbA1c <7.0  30 Diabetes with HbA1c 7.0–7.9  20 Diabetes with HbA1c 8.0–8.9  10 Diabetes with Hb A1c 9.0–9.9  0 Diabetes with HbA1c ≥10.0 |  |
| Blood pressure (BP) | Measurement: Appropriately measured systolic and diastolic blood pressures (Average values of systolic and diastolic BP blood pressure were utilized, and automated readings were preferred. In case where automated readings were unavailable, manual readings were employed.) | Metric: Systolic and diastolic BPs (mm Hg)  Scoring:  Points Level  100 <120/<80 (optimal)  75 120–129/<80 (elevated)  50 130–139 or 80–89 (stage 1 hypertension)  25 140–159 or 90–99  0 ≥160 or ≥100  Subtract 20 points if treated level |  |

**Supplementary Table 2.** Healthy Diet Score using touchscreen questionnaire in the UK biobank study.

| **Food group/nutrient item** | **UKB fields used** | **Definition of meeting criterion** | **Amount per serving** |
| --- | --- | --- | --- |
| **Consume more Fruits, fresh or dried** | 1309 and 1319 | ≥3 servings/d  Including fresh and dried fruits | 1309 – 1 piece  1319 – 3 pieces |
| **Vegetables, salad/cooked** | 1289 and 1299 | ≥3 servings/d  Including salad, raw and cooked | 1289 – 8 tablespoons  1299 – 12 tablespoons |
| **Whole grains** | 1438 and 1448  1458 and 1468 | ≥3 servings/d  Daily slices of whole meal or wholegrain bread (servings/d), convert from weekly slices.  Daily bowls of whole wheat cereal as servings/d (bran cereal, biscuit cereal, oat cereal and muesli), convert from weekly bowls. | 1438/1448 – 1 slice/d  1458/1468 – 1 bowl/d |
| **Fish shellfish** | 1329 and 1339 | ≥2 servings/wk. | Once/wk. |
| **Dairy products** | 6144, 1408 and 1418 | Sum weekly frequencies to obtain total servings/wk.  Reporting consumption of one milk item or eating cheese once a  day to meet the 2-3 servings/d criterion. | 1408 – once/d  1418 – consumption of any type of milk |
| **Vegetable oil** | 2654 | Reporting use of olive oil or polyunsaturated/sunflower oil (yes=1, 0=no) |  |
| **Consume less Refined grains, starches, added sugars** | 1438 and 1448  1458 and 1468 | Reporting use of white, brown, other bread or other cereals; <1.5 servings/d | 1438/1448 – 1 slice/d  1458/1468 – 1 bowl/d |
| **Processed meats** | 1349 and 3680 | Once/wk. or less would meet the criterion. |  |
| **Unprocessed red meats** | 1369, 1379, 1389, and 3680 | Summation of frequency of consumption across three types of red meats (lamb/mutton, beef or pork). <3 on the summation corresponds to the criterion of <1-2 9 servings per week. |  |
| **Industrial trans fat** | 1428 and 2654 | Reporting use of Flora Pro-Active/Benaco spread, soft margarine -, olive oil based -, polyunsaturated/ sunflower oil based -, other low/reduced fat spread or never use spread would meet the criterion |  |
| **Sugar-sweetened beverages** | 6144 | Never eat sugar or food/drink containing sugar would meet the criterion |  |
| **Sodium** | 1478 | Salt added to food, never or rarely would meet the criterion |  |

**Supplementary Table 3. Single nucleotide polymorphisms (SNPs) used to build the GRS in the UK Biobank Study for hip and/or knee OA.**

|  | | | | | | | | | | |
| --- | --- | --- | --- | --- | --- | --- | --- | --- | --- | --- |
| SNP | CHR: POS | EA | NEA | EAF | Hip/knee OA | | Hip OA | | Knee OA | |
|  |  |  |  |  | OR | *P* | OR | *P* | OR | *P* |
| rs11164653 | 1:103464210 | T | C | 0.41 |  |  | 0.923208663 | 2.77E-18 |  |  |
| rs2622873 | 1:103466053 | T | C | 0.88 | 1.057280452 | 2.30E-09 |  |  | 1.029733468 | 5.86E-03 |
| rs4411121 | 1:118757034 | T | C | 0.31 | 1.022243784 | 7.54E-04 | 1.066945614 | 2.16E-11 |  |  |
| rs10797923 | 1:183901966 | T | C | 0.69 | 1.028807125 | 1.83E-05 | 1.043207394 | 1.57E-05 | 1.028498529 | 2.69E-04 |
| rs1046934 | 1:184023529 | A | C | 0.65 | 1.035826853 | 2.93E-08 | 1.073795964 | 3.81E-14 | 1.021834969 | 3.33E-03 |
| rs2605098 | 1:219643649 | A | G | 0.33 | 1.051165975 | 7.25E-15 | 1.067265746 | 6.83E-12 |  |  |
| rs2791549 | 1:219658003 | A | C | 0.30 |  |  |  |  | 1.046760336 | 3.06E-09 |
| rs3740129 | 10:73767859 | A | G | 0.46 |  |  | 1.0562237 | 1.84E-09 | 0.987479048 | 7.78E-02 |
| rs10824456 | 10:78615458 | C | G | 0.58 | 0.968119257 | 1.63E-07 | 0.968700303 | 4.84E-04 | 0.971610767 | 6.57E-05 |
| rs3993110 | 11:12794530 | A | C | 0.61 |  |  |  |  | 1.031279227 | 1.67E-05 |
| rs11022512 | 11:12835952 | A | G | 0.41 |  |  | 1.045818675 | 1.01E-06 |  |  |
| rs1517572 | 11:28829882 | A | C | 0.41 | 1.038939 | 6.79E-10 |  |  | 1.03334385 | 5.19E-06 |
| rs2959129 | 11:28851988 | C | G | 0.43 |  |  | 1.044355554 | 1.84E-06 |  |  |
| rs1631174 | 11:47974373 | A | C | 0.34 | 1.03956255 | 7.28E-09 | 1.050850672 | 4.30E-07 | 1.026443588 | 9.11E-04 |
| rs67924081 | 11:65342981 | A | G | 0.74 | 1.02408548 | 6.72E-04 | 1.066305639 | 7.80E-10 |  |  |
| rs34560402 | 11:66872320 | T | C | 0.06 | 0.932020937 | 1.49E-07 | 0.887452749 | 1.59E-09 | 0.963387076 | 1.82E-02 |
| rs72979233 | 11:74355523 | A | G | 0.75 | 0.964447385 | 4.14E-07 |  |  | 0.951609992 | 2.54E-09 |
| rs1149620 | 11:76506572 | A | T | 0.44 | 0.962712941 | 2.87E-09 | 0.962712941 | 4.95E-05 | 0.962809217 | 3.98E-07 |
| rs10831475 | 11:95796907 | A | G | 0.81 |  |  |  |  | 1.038835111 | 2.39E-05 |
| rs10831476 | 11:95796910 | A | C | 0.81 | 1.045609532 | 9.53E-09 |  |  |  |  |
| rs10831477 | 11:95797111 | T | G | 0.81 |  |  | 1.07293727 | 1.20E-09 |  |  |
| rs1426371 | 12:108629780 | A | G | 0.27 | 0.960021115 | 1.34E-08 |  |  | 0.94980365 | 8.86E-10 |
| rs753350451 | 12:123732769 | D | I | 0.20 |  |  |  |  | 0.93220736 | 3.36E-10 |
| rs1809889 | 12:124801226 | T | C | 0.28 | 1.033240521 | 1.50E-06 | 1.061411897 | 3.58E-09 | 1.023675928 | 3.11E-03 |
| rs7294636 | 12:15054016 | A | G | 0.37 |  |  |  |  | 0.978827355 | 3.50E-03 |
| rs10842226 | 12:23959589 | A | G | 0.42 | 1.042477404 | 4.68E-10 | 1.047283847 | 3.48E-06 | 1.04780762 | 3.63E-09 |
| rs10492367 | 12:28014970 | T | G | 0.19 |  |  | 1.121536926 | 2.75E-24 |  |  |
| rs10843013 | 12:28025196 | A | C | 0.78 | 0.95341977 | 1.42E-10 |  |  |  |  |
| rs7967762 | 12:48420214 | T | C | 0.17 | 1.044877862 | 9.61E-08 |  |  | 1.05865581 | 2.09E-09 |
| rs79056043 | 12:59289598 | A | G | 0.94 | 0.961077719 | 2.37E-03 | 0.89261493 | 1.99E-09 |  |  |
| rs7953280 | 12:94136009 | C | G | 0.50 | 1.043103079 | 4.84E-12 | 1.038004375 | 3.62E-05 | 1.033964042 | 2.70E-06 |
| rs58973023 | 13:42959133 | A | T | 0.49 | 1.04226893 | 2.37E-08 |  |  | 1.057069017 | 4.72E-10 |
| rs28929474 | 14:94844947 | T | C | 0.02 | 0.911922747 | 7.50E-05 | 0.884705905 | 3.75E-04 | 0.916035646 | 1.18E-03 |
| rs4380013 | 15:50759428 | A | G | 0.19 | 1.048960842 | 1.24E-09 |  |  | 1.057809224 | 8.73E-10 |
| rs4775006 | 15:58215727 | A | C | 0.42 | 1.026340948 | 5.79E-05 |  |  | 1.047493324 | 8.55E-10 |
| rs746239049 | 15:63067433 | D | I | 0.21 |  |  | 0.91860414 | 3.35E-10 |  |  |
| rs12908498 | 15:67366488 | C | G | 0.54 |  |  | 1.083070432 | 1.85E-16 |  |  |
| rs12901372 | 15:67370506 | C | G | 0.53 | 1.046865017 | 6.09E-13 |  |  | 1.03603404 | 1.96E-06 |
| rs12914479 | 15:99174828 | C | G | 0.66 | 1.035619709 | 7.38E-08 |  |  | 1.044773379 | 7.12E-09 |
| rs6500609 | 16:4515334 | C | G | 0.11 | 0.948664569 | 8.40E-08 |  |  | 0.935382259 | 5.16E-09 |
| rs9940278 | 16:53800200 | T | C | 0.43 | 1.055168004 | 1.45E-18 | 1.055801295 | 1.77E-09 | 1.059714996 | 3.19E-16 |
| rs34195470 | 16:69955690 | A | G | 0.45 | 0.969960432 | 8.02E-07 |  |  | 0.949044111 | 3.13E-13 |
| rs216175 | 17:2167690 | A | C | 0.83 | 1.04780762 | 8.22E-09 |  |  | 1.048960842 | 3.86E-07 |
| rs4548913 | 17:2209888 | A | G | 0.63 | 0.960885523 | 2.32E-10 |  |  | 0.950278671 | 3.15E-12 |
| rs227732 | 17:54769890 | T | C | 0.30 | 1.045191372 | 3.97E-08 | 1.058020807 | 1.05E-06 | 1.050535464 | 3.37E-07 |
| rs1401796 | 17:54839759 | A | C | 0.51 |  |  | 0.943366895 | 1.43E-10 |  |  |
| rs9908159 | 17:54841961 | T | C | 0.51 | 1.04091486 | 4.44E-11 |  |  | 1.03468807 | 1.45E-06 |
| rs7222178 | 17:59652282 | A | T | 0.19 | 1.024290318 | 1.90E-03 | 1.068867846 | 7.35E-09 |  |  |
| rs2521348 | 17:67499717 | T | C | 0.39 |  |  | 1.057069017 | 1.56E-09 |  |  |
| rs2716212 | 17:67503653 | A | G | 0.62 | 0.965605416 | 1.67E-08 |  |  | 0.970930878 | 4.22E-05 |
| rs2163832 | 19:10745764 | T | C | 0.32 | 1.034481153 | 2.47E-07 |  |  | 1.046446355 | 2.73E-09 |
| rs75621460 | 19:41833784 | A | G | 0.03 | 1.11627807 | 3.61E-07 | 1.177272121 | 3.25E-07 | 1.097900808 | 2.29E-04 |
| rs8112559 | 19:46390455 | C | G | 0.89 |  |  |  |  | 0.97902314 | 5.32E-02 |
| rs4252548 | 19:55879672 | T | C | 0.02 | 1.07079354 | 4.51E-04 | 1.252823745 | 2.24E-15 |  |  |
| rs551471509 | 19:9943264 | T | C | 1.00 | 0.175836622 | 1.15E-08 |  |  |  |  |
| rs66989638 | 2:106689736 | A | G | 0.13 | 1.030145444 | 1.31E-03 | 1.082096107 | 4.79E-09 |  |  |
| rs62182810 | 2:204387482 | A | G | 0.54 | 1.021222052 | 6.62E-04 |  |  | 1.028807125 | 7.52E-05 |
| rs7581446 | 2:33423801 | T | C | 0.48 | 0.952657339 | 4.87E-11 | 0.955806301 | 2.07E-05 | 0.945066484 | 1.71E-10 |
| rs66906321 | 2:630070 | T | C | 0.18 |  |  |  |  | 0.945066484 | 1.71E-09 |
| rs74676797 | 2:633063 | A | G | 0.82 | 1.051060863 | 6.39E-10 | 1.04331172 | 3.83E-04 |  |  |
| rs2862851 | 2:70712802 | T | C | 0.47 |  |  | 1.067692737 | 3.86E-13 |  |  |
| rs3755381 | 2:70718695 | T | C | 0.47 | 1.045191372 | 4.23E-13 |  |  | 1.034067444 | 2.19E-06 |
| rs143384 | 20:34025756 | A | G | 0.59 | 1.059397129 | 1.24E-20 |  |  | 1.074870297 | 1.01E-23 |
| rs9981408 | 21:40017446 | T | G | 0.23 | 1.024700116 | 7.16E-04 | 1.059185271 | 5.99E-08 |  |  |
| rs9981884 | 21:40585633 | A | G | 0.49 | 0.966958211 | 3.27E-08 | 0.956284324 | 6.38E-07 | 0.967248342 | 2.44E-06 |
| rs11705555 | 22:28206912 | A | C | 0.76 | 1.035930441 | 1.79E-06 |  |  | 1.052533379 | 2.99E-09 |
| rs12160491 | 22:38195796 | A | G | 0.71 | 0.967054911 | 4.23E-07 | 0.940446986 | 4.37E-10 | 0.978729477 | 4.98E-03 |
| rs2276749 | 3:11643465 | T | C | 0.05 | 0.951990713 | 3.91E-04 | 0.890208121 | 1.97E-08 | 0.978044607 | 1.63E-01 |
| rs781661531 | 3:187051013 | T | C | 1.00 | 0.415073361 | 2.86E-04 | 0.105030972 | 8.36E-11 | 0.623504061 | 1.05E-01 |
| rs747952496 | 3:188311659 | A | G | 0.00 | 2.555633147 | 1.83E-07 | 7.019556223 | 4.91E-11 | 2.364106147 | 4.63E-04 |
| rs9835230 | 3:189735461 | A | G | 0.24 | 1.03738176 | 2.45E-07 | 1.065879202 | 1.34E-09 | 1.026546237 | 1.55E-03 |
| rs62242105 | 3:20630395 | A | G | 0.33 | 0.976969349 | 3.55E-04 |  |  | 0.970930878 | 1.04E-04 |
| rs1546737 | 3:52407041 | T | C | 0.33 | 1.040290499 | 1.26E-09 |  |  | 1.025007572 | 1.10E-03 |
| rs2268023 | 3:52819327 | A | T | 0.41 |  |  | 1.070151257 | 1.56E-13 |  |  |
| rs6855246 | 4:103112470 | A | G | 0.93 |  |  | 0.896999477 | 7.94E-09 |  |  |
| rs13107325 | 4:103188709 | T | C | 0.07 | 1.076591466 | 1.83E-09 |  |  | 1.067265746 | 4.95E-06 |
| rs11729628 | 4:121584282 | T | G | 0.24 | 0.96908786 | 1.15E-05 |  |  | 0.963098103 | 6.69E-06 |
| rs1913707 | 4:13039440 | A | G | 0.60 | 1.042790195 | 1.46E-11 | 1.070044247 | 1.82E-13 | 1.02716235 | 2.06E-04 |
| rs75686861 | 4:145621328 | A | G | 0.09 | 1.039666512 | 2.95E-04 | 1.086650468 | 1.24E-07 |  |  |
| rs798756 | 4:1707447 | T | C | 0.19 |  |  | 0.933980237 | 2.24E-09 |  |  |
| rs11731421 | 4:1749160 | A | G | 0.35 | 1.039458599 | 1.94E-09 |  |  |  |  |
| rs7680647 | 4:1750487 | T | C | 0.63 |  |  |  |  | 0.956762586 | 1.24E-08 |
| rs59163323 | 4:1763318 | A | G | 0.20 | 0.945350047 | 2.92E-12 |  |  | 0.94980365 | 5.03E-08 |
| rs201194999 | 4:66666895 | T | C | 0.30 | 0.882849972 | 1.16E-07 | 0.873104525 | 3.43E-06 | 0.896192541 | 7.16E-06 |
| rs1560080 | 5:115338732 | A | G | 0.83 |  |  |  |  | 0.966474852 | 2.88E-04 |
| rs17677724 | 5:128015370 | T | C | 0.16 |  |  | 1.074977789 | 3.54E-09 |  |  |
| rs17615906 | 5:128018413 | T | C | 0.84 | 0.94714792 | 3.76E-11 |  |  | 0.949044111 | 4.88E-08 |
| rs10062749 | 5:141805088 | T | G | 0.27 |  |  |  |  |  |  |
| rs10038860 | 5:141809938 | A | G | 0.27 | 1.033964042 | 9.24E-07 |  |  | 1.047074411 | 5.62E-09 |
| rs4073717 | 5:170864021 | T | G | 0.20 |  |  | 0.935008181 | 2.54E-09 |  |  |
| rs3884606 | 5:170871074 | A | G | 0.52 | 0.963290742 | 8.96E-10 |  |  | 0.972388367 | 7.76E-05 |
| rs2066928 | 5:30843787 | A | G | 0.48 | 0.970154444 | 1.00E-06 |  |  | 0.959637183 | 1.20E-08 |
| rs10940168 | 5:67823586 | A | G | 0.39 |  |  | 0.948000736 | 7.74E-09 |  |  |
| rs56132153 | 5:67825133 | A | C | 0.61 |  |  |  |  | 0.985900344 | 5.04E-02 |
| rs9396861 | 6:18404133 | A | C | 0.61 |  |  |  |  | 0.984914937 | 7.60E-02 |
| rs79220007 | 6:26098474 | T | C | 0.93 | 0.948285179 | 1.20E-05 | 0.899514595 | 2.22E-09 | 0.966571505 | 1.80E-02 |
| rs2856821 | 6:33046742 | T | C | 0.79 | 1.047179124 | 5.71E-09 | 1.048436493 | 3.76E-05 | 1.045714099 | 1.10E-06 |
| rs2038740 | 6:35114542 | T | C | 0.72 | 0.969766459 | 5.94E-06 | 0.950183647 | 4.14E-07 | 0.976188086 | 2.22E-03 |
| rs6908606 | 6:44998888 | A | G | 0.71 |  |  | 0.933513364 | 3.86E-12 |  |  |
| rs12193102 | 6:45145402 | C | G | 0.61 | 0.948854321 | 3.50E-09 |  |  | 0.964061683 | 8.38E-04 |
| rs80287694 | 6:55636940 | A | G | 0.89 | 0.953896599 | 1.39E-06 |  |  | 0.972680127 | 1.51E-02 |
| rs9475400 | 6:55638258 | T | C | 0.10 |  |  | 1.114493454 | 8.03E-13 |  |  |
| rs12209223 | 6:76164589 | A | C | 0.11 | 1.051902048 | 2.64E-07 | 1.150043767 | 1.88E-22 |  |  |
| rs116934101 | 7:101775597 | A | G | 0.27 | 1.042894479 | 7.12E-07 | 1.052954476 | 2.19E-05 | 1.036759517 | 5.22E-04 |
| rs12667224 | 7:114024316 | A | G | 0.52 | 0.976773975 | 1.11E-04 |  |  | 0.972874683 | 9.91E-05 |
| rs143083812 | 7:128843410 | T | C | 0.00 | 1.920719778 | 2.29E-07 | 2.896201353 | 8.21E-11 |  |  |
| rs571734653 | 7:137143697 | A | C | 0.00 | 6.030922596 | 5.56E-09 | 9.023208677 | 4.33E-08 | 5.764970289 | 4.25E-06 |
| rs7787744 | 7:150521096 | A | G | 0.67 | 1.027778832 | 1.84E-05 |  |  | 1.039146808 | 2.39E-07 |
| rs111844273 | 7:18436337 | A | G | 0.02 | 1.101640024 | 1.48E-05 | 1.26074145 | 1.05E-12 |  |  |
| rs10956488 | 8:130717755 | A | G | 0.84 |  |  |  |  | 1.019385505 | 4.84E-02 |
| rs765002298 | 8:130733847 | D | I | 0.20 |  |  | 0.899154861 | 1.75E-15 |  |  |
| rs80308593 | 9:110403802 | C | G | 0.85 | 1.059185271 | 1.90E-11 |  |  | 1.042998773 | 2.34E-05 |
| rs79895530 | 9:110416422 | T | C | 0.13 |  |  | 0.904385112 | 7.04E-14 |  |  |
| rs72760655 | 9:116916214 | A | C | 0.33 | 1.047598079 | 5.97E-13 |  |  | 1.050115334 | 7.25E-11 |
| rs737142 | 9:116929327 | T | C | 0.67 |  |  | 0.957049658 | 3.73E-06 |  |  |
| rs1330349 | 9:117840742 | C | G | 0.59 | 1.021222052 | 9.97E-04 | 1.064920342 | 6.94E-12 |  |  |
| rs7862601 | 9:118343026 | A | G | 0.62 | 0.96608834 | 5.42E-06 | 0.938661833 | 6.19E-09 | 0.981179362 | 3.76E-02 |
| rs1321917 | 9:119324929 | C | G | 0.41 |  |  |  |  | 0.990346893 | 1.76E-01 |
| rs2416564 | 9:119370679 | T | C | 0.60 | 0.980296698 | 1.26E-03 | 0.929229064 | 1.00E-15 |  |  |
| rs10983775 | 9:120521100 | T | C | 0.54 | 0.970833789 | 1.29E-06 | 0.948380012 | 4.65E-09 | 0.978044607 | 1.87E-03 |
| rs12377624 | 9:129373110 | C | G | 0.36 |  |  |  |  | 1.025622761 | 6.50E-04 |
| rs62578126 | 9:129375338 | T | C | 0.37 |  |  | 0.939319126 | 4.29E-11 |  |  |
| rs10465114 | 9:129917824 | A | G | 0.22 | 1.030145444 | 4.96E-05 | 1.064494459 | 9.04E-09 |  |  |
| rs10974438 | 9:4291928 | A | C | 0.65 | 1.042581657 | 7.39E-11 | 1.040810774 | 2.76E-05 | 1.044355554 | 4.89E-09 |
| rs76340814 | 9:98321412 | A | G | 0.05 | 0.955137471 | 8.83E-04 | 0.931089382 | 4.85E-04 | 0.963772508 | 2.07E-02 |
| rs148693048 | 8:24598320 | T | C | 0.00 |  |  |  |  | 1.156617735 | 1.63E-01 |
| Abbreviation: EA, effect allele; NEA, non-effect allele; EAF, weighted average of effect allele frequency across all studies; OA, osteoarthritis | | | | | | | | | | |

| Supplementary Table 4. Associations Between Components of LE8 and OA^a^. | | | |
| --- | --- | --- | --- |
|  | Osteoarthritis of hip and/or knee | Osteoarthritis of hip | Osteoarthritis of knee |
|  | HR (95% CI) ^b^ | HR (95% CI) | HR (95% CI) |
| Diet score | | | |
| Low | 1.00 (reference) | 1.00 (reference) | 1.00 (reference) |
| Moderate | 0.95 (0.91, 0.98) | 1.02 (0.97, 1.08) | 0.91 (0.87, 0.95) |
| High | 0.94 (0.90, 0.97) | 1.02 (0.96, 1.09) | 0.89 (0.85, 0.94) |
| Physical activity score |  |  |  |
| Low | 1.00 (reference) | 1.00 (reference) | 1.00 (reference) |
| Moderate | 0.97 (0.91, 1.04) | 0.93 (0.83, 1.03) | 1.00 (0.92, 1.09) |
| High | 1.07 (1.04, 1.11) | 1.05 (1.00, 1.11) | 1.09 (1.04, 1.14) |
| Nicotine exposure score |  |  |  |
| Low | 1.00 (reference) | 1.00 (reference) | 1.00 (reference) |
| Moderate | 1.10 (1.05, 1.16) | 0.99 (0.91, 1.07) | 1.21 (1.13, 1.29) |
| High | 1.03 (0.98, 1.09) | 0.83 (0.83, 0.98) | 1.14 (1.07, 1.22) |
| Sleep health score |  |  |  |
| Low | 1.00 (reference) | 1.00 (reference) | 1.00 (reference) |
| Moderate | 0.88 (0.83, 0.94) | 0.86 (0.78, 0.95) | 0.89 (0.83, 0.96) |
| High | 0.77 (0.73, 0.81) | 0.79 (0.72, 0.86) | 0.75 (0.70, 0.80) |
| Body mass index score |  |  |  |
| Low (high BMI) | 1.00 (reference) | 1.00 (reference) | 1.00 (reference) |
| Moderate | 0.61 (0.59, 0.63) | 0.78 (0.74, 0.82) | 0.53 (0.51, 0.55) |
| High (low BMI) | 0.37 (0.36, 0.39) | 0.59 (0.56, 0.63) | 0.27 (0.26, 0.29) |
| Blood lipids score |  |  |  |
| Low (high non-HDL) | 1.00 (reference) | 1.00 (reference) | 1.00 (reference) |
| Moderate | 0.99 (0.95, 1.02) | 0.99 (0.94, 1.05) | 0.99 (0.94, 1.03) |
| High (low non-HDL) | 0.99 (0.95, 1.03) | 0.99 (0.93, 1.04) | 1.00 (0.96, 1.05) |
| Blood glucose score |  |  |  |
| Low (high glucose/Hbalc) | 1.00 (reference) | 1.00 (reference) | 1.00 (reference) |
| Moderate | 0.95 (0.52, 1.72) | 0.25 (0.04, 1.78) | 1.26 (0.67, 2.34) |
| High (low glucose/Hbalc) | 0.88 (0.82, 0.93) | 1.00 (0.90, 1.11) | 0.81 (0.76, 0.87) |
| Blood pressure score |  |  |  |
| Low (high BP) | 1.00 (reference) | 1.00 (reference) | 1.00 (reference) |
| Moderate | 0.90 (0.87, 0.93) | 0.94 (0.90, 0.99) | 0.87 (0.83, 0.91) |
| High (low BP) | 0.84 (0.80, 0.88) | 0.92 (0.85, 0.99) | 0.79 (0.75, 0.84) |
| a Abbreviations: LE8, Life’s Essential 8 ; OA, Osteoarthritis. | | | |
| b Hazard ratio (95% confidence interval) (all such values). | | | |
| c Multivariable Cox proportional regression was adjusted for age (continuous), sex (male or female), race (White, Asian, Black, or others), Townsend deprivation index (categorical, quartiles), drinking status (current, previous, or never), and education levels (low, moderate, or high). | | | |

|  | | | | |
| --- | --- | --- | --- | --- |
| Supplementary Table 5 **Associations of GRS with the risks of the osteoarthritis (n = 242,278) a** | | | |  |
|  | GRS | | |  |
|  | < Median | ≥Moderate | Each SD increase |  |
| Osteoarthritis of hip and/or knee | | | | |
| Cases, n | 8,419 | 10,348 | 18,767 |  |
| Person-years | 1,374,573 | 1,363,473 | 2,738,046 |  |
| Model 1 c | 1.00 (reference) | 1.24 (1.21, 1.28) b | 1.14 (1.12, 1.15) |  |
| Model 2 d | 1.00 (reference) | 1.26 (1.22, 1.29) | 1.15 (1.13, 1.16) |  |
| Model 3 e | 1.00 (reference) | 1.25 (1.22, 1.29) | 1.14 (1.13, 1.16) |  |
|  |  |  |  |  |
| Osteoarthritis of hip |  |  |  |  |
| Cases, n | 3,135 | 4,583 | 7,718 |  |
| Person-years | 1,406,596 | 1,398,589 | 2,805,185 |  |
| Model 1 c | 1.00 (reference) | 1.47 (1.41, 1.54) | 1.24 (1.22, 1.27) |  |
| Model 2 d | 1.00 (reference) | 1.48 (1.42, 1.55) | 1.25 (1.22, 1.27) |  |
| Model 3 e | 1.00 (reference) | 1.47 (1.41, 1.54) | 1.24 (1.22, 1.27) |  |
|  |  |  |  |  |
| Osteoarthritis of knee |  |  |  |  |
| Cases, n | 5,290 | 6,597 | 11,887 |  |
| Person-years | 1,391,081 | 1,384,572 | 2,775,653 |  |
| Model 1 c | 1.00 (reference) | 1.25 (1.21, 1.30) | 1.14 (1.12, 1.16) |  |
| Model 2 d | 1.00 (reference) | 1.26 (1.22, 1.31) | 1.15 (1.13, 1.17) |  |
| Model 3 e | 1.00 (reference) | 1.26 (1.21, 1.30) | 1.15 (1.13, 1.17) |  |
| a Abbreviations: GRS, **Genetic** risk scores; SD, standard deviation. | | | | |
| b Hazard ratio (95% confidence interval) (all such values). | | | | |
| c Model 1 was a crude model. | | | | |
| d Model 2 was adjusted for age (continuous) and sex (male or female). | | | | |
| e Model 3 was further adjusted for race (White, Asian, Black, or others), Townsend deprivation index (categorical, quartiles), drinking status (current, previous, or never), education levels (low, moderate, or high), and Life's Essential 8 scores. | | | |  |
|  |  |  |  |  |
|  |  |  |  |  |

Supplementary Table 6. Associations Between Components of LE8 and Osteoarthritis stratifying by GRS.

|  | **Low GRS** | | | **High GRS** | | |
| --- | --- | --- | --- | --- | --- | --- |
|  | Osteoarthritis of hip and/or knee | Osteoarthritis of hip | Osteoarthritis of knee | Osteoarthritis of hip and/or knee | Osteoarthritis of hip | Osteoarthritis of knee |
|  | HR (95% CI) ^b^ | HR (95% CI) | HR (95% CI) | HR (95% CI) ^b^ | HR (95% CI) | HR (95% CI) |
| Diet score |  |  |  |  |  |  |
| Low | 1.00 (reference) ^c^ | 1.00 (reference) | 1.00 (reference) | 1.00 (reference) | 1.00 (reference) | 1.00 (reference) |
| Moderate | 0.95 (0.91, 0.98) | 0.97 (0.89, 1.06) | 0.90 (0.85, 0.97) | 0.97 (0.93, 1.02) | 1.07 (1.00, 1.15) | 0.91 (0.86, 0.96) |
| High | 0.94 (0.90, 0.97) | 0.98 (0.90, 1.07) | 0.90 (0.84, 0.97) | 0.96 (0.91, 1.01) | 1.06 (0.98, 1.15) | 0.89 (0.83, 0.95) |
| *P* for interaction | 0.2368 | 0.1798 | 0.6727 | 0.2368 | 0.1798 | 0.6727 |
| Physical activity score |  |  |  |  |  |  |
| Low | 1.00 (reference) | 1.00 (reference) | 1.00 (reference) | 1.00 (reference) | 1.00 (reference) | 1.00 (reference) |
| Moderate | 1.05 (0.95, 1.16) | 0.93 (0.79, 1.11) | 1.00 (0.88, 1.14) | 0.91 (0.83, 0.99) | 0.92 (0.80, 1.05) | 0.99 (0.89, 1.11) |
| High | 1.12 (1.06, 1.18) | 1.11 (1.02, 1.21) | 1.11 (1.04, 1.19) | 1.04 (0.99, 1.09) | 1.02 (0.94, 1.09) | 1.07 (1.01, 1.14) |
| *P* for interaction | 0.0601 | 0.1264 | 0.3297 | 0.0601 | 0.1264 | 0.3297 |
| Nicotine exposure score |  |  |  |  |  |  |
| Low | 1.00 (reference) | 1.00 (reference) | 1.00 (reference) | 1.00 (reference) | 1.00 (reference) | 1.00 (reference) |
| Moderate | 1.13 (1.05, 1.23) | 1.08 (0.95, 1.22) | 1.19 (1.07, 1.31) | 1.09 (1.02, 1.17) | 0.94 (0.85, 1.04) | 1.22 (1.11, 1.33) |
| High | 1.08 (1.00, 1.18) | 0.97 (0.85, 1.10) | 1.14 (1.03, 1.27) | 0.99 (0.93, 1.07) | 0.86 (0.78, 0.96) | 1.14 (1.04, 1.24) |
| *P* for interaction | 0.1314 | 0.6282 | 0.8508 | 0.1314 | 0.6282 | 0.8508 |
| Sleep health score |  |  |  |  |  |  |
| Low | 1.00 (reference) | 1.00 (reference) | 1.00 (reference) | 1.00 (reference) | 1.00 (reference) | 1.00 (reference) |
| Moderate | 0.90 (0.82, 0.98) | 0.87 (0.75, 1.01) | 0.91 (0.81, 1.01) | 0.87 (0.80, 0.94) | 0.86 (0.76, 0.97) | 0.88 (0.80, 0.98) |
| High | 0.77 (0.71, 0.83) | 0.81 (0.71, 0.93) | 0.73 (0.66, 0.81) | 0.76 (0.71, 0.82) | 0.77 (0.69, 0.86) | 0.76 (0.69, 0.83) |
| *P* for interaction | 0.7426 | 0.5696 | 0.2249 | 0.7426 | 0.5696 | 0.2249 |
| Body mass index score |  |  |  |  |  |  |
| Low (high BMI) | 1.00 (reference) | 1.00 (reference) | 1.00 (reference) | 1.00 (reference) | 1.00 (reference) | 1.00 (reference) |
| Moderate | 0.59 (0.56, 0.61) | 0.78 (0.72, 0.85) | 0.51 (0.49, 0.55) | 0.63 (0.30, 0.66) | 0.78 (0.72, 0.83) | 0.55 (0.52, 0.58) |
| High (low BMI) | 0.36 (0.34, 0.38) | 0.60 (0.55, 0.66) | 0.26 (0.24, 0.29) | 0.39 (0.37, 0.41) | 0.59 (0.55, 0.64) | 0.28 (0.26, 0.30) |
| *P* for interaction | 0.0120 | 0.9380 | 0.0961 | 0.0120 | 0.9380 | 0.0961 |
| Blood lipids score |  |  |  |  |  |  |
| Low (high non-HDL) | 1.00 (reference) | 1.00 (reference) | 1.00 (reference) | 1.00 (reference) | 1.00 (reference) | 1.00 (reference) |
| Moderate | 0.96 (0.91, 1.01) | 0.97 (0.89, 1.07) | 0.98 (0.91, 1.05) | 1.01 (0.96, 1.06) | 1.01 (0.93, 1.08) | 1.00 (0.94, 1.06) |
| High (low non-HDL) | 0.98 (0.93, 1.04) | 0.98 (0.90, 1.07) | 0.97 (0.91, 1.04) | 0.99 (0.95, 1.04) | 0.99 (0091, 1.06) | 1.03 (0.97, 1.09) |
| *P* for interaction | 0.5802 | 0.8799 | 0.2414 | 0.5802 | 0.8799 | 0.2414 |
| Blood glucose score |  |  |  |  |  |  |
| Low (high glucose/Hbalc) | 1.00 (reference) | 1.00 (reference) | 1.00 (reference) | 1.00 (reference) | 1.00 (reference) | 1.00 (reference) |
| Moderate | 1.52 (0.76, 3.06) | 0.34 (0.21, 0.55) | 2.35 (1.17, 4.73) | 0.48 (0.15, 1.48) | 0.44 (0.06, 3.16) | 0.43. (0.11, 1.73) |
| High (low glucose/Hbalc) | 0.92 (0.84, 1.01) | 1.03 (0.88, 1.21) | 0.80 (0.71, 0.89) | 0.85 (0.78, 0.92) | 0.98 (0.86, 1.12) | 0.83 (0.75, 0.92) |
| *P* for interaction | 0.3735 | 0.7782 | 0.3307 | 0.3735 | 0.7782 | 0.3307 |
| Blood pressure score |  |  |  |  |  |  |
| Low (high BP) | 1.00 (reference) | 1.00 (reference) | 1.00 (reference) | 1.00 (reference) | 1.00 (reference) | 1.00 (reference) |
| Moderate | 0.90 (0.86, 0.94) | 1.00 (0.92, 1.08) | 0.86 (0.81, 0.91) | 0.90 (0.86, 0.94) | 0.91 (0.85, 0.97) | 0.88 (0.84, 0.93) |
| High (low BP) | 0.83 (0.77, 0.89) | 0.91 (0.81, 1.03) | 0.81 (0.74, 0.89) | 0.85 (0.80, 0.90) | 0.93 (0.85, 1.02) | 0.78 (0.72, 0.85) |
| *P* for interaction | 0.3342 | 0.6366 | 0.5274 | 0.3342 | 0.6366 | 0.5274 |
| a Abbreviations: LE8, Life’s Essential 8 ; OA, Osteoarthritis. | | | | | | |
| b Hazard ratio (95% confidence interval) (all such values). | | | | | | |
| c Multivariable Cox proportional regression was adjusted for age (continuous), sex (male or female), race (White, Asian, Black, or others), Townsend deprivation index (categorical, quartiles), drinking status (current, previous, or never), education levels (low, moderate, or high), genotyping batch, and the first 10 principal components of genetics | | | | | | |

| Supplementary **Table 7**. Sensitivity analyses of the association between LE8 score and the risk of osteoarthritis ^a^ | | | | | | | | |
| --- | --- | --- | --- | --- | --- | --- | --- | --- |
|  | | Hazard ratio (95% confidence interval) ^b^ | | | | Each SD increase in LE8 score | Each SD increase in behavior scale | Each SD increase in biological scale |
|  |  | Low LE8  (0-49) | Moderate LE8 (50-79) | | High LE8  (80-100) |  |  |  |
| 1) excluding osteoarthritis cases diagnosed in the first 1 year of follow-up (n = 241,321) | | | | | | | | |
| Osteoarthritis of hip and/or knee | 1.00 (reference) | | | 0.77 (0.73, 0.82) | 0.56 (0.52, 0.60) | 0.84 (0.83, 0.86) | 0.85 (0.83, 0.86) | 0.94 (0.92, 0.95) |
| Osteoarthritis of hip | 1.00 (reference) | | | 0.82 (0.74, 0.90) | 0.72 (0.64, 0.81) | 0.91 (0.89, 0.93) | 0.91 (0.89, 0.93) | 0.97 (0.95, 0.99) |
| Osteoarthritis of knee | 1.00 (reference) | | | 0.76 (0.70, 0.81) | 0.48 (0.43, 0.52) | 0.80 (0.79, 0.82) | 0.81 (0.80, 0.83) | 0.91 (0.89, 0.93) |
|  |  | | |  |  |  |  |  |
| 2) excluding osteoarthritis cases diagnosed in the first 2 year of follow-up (n = 240,038) | | | | | | | | |
| Osteoarthritis of hip and/or knee | 1.00 (reference) | | | 0.77 (0.72, 0.82) | 0.56 (0.52, 0.60) | 0.84 (0.83, 0.86) | 0.84 (0.83, 0.86) | 0.93 (0.92, 0.95) |
| Osteoarthritis of hip | 1.00 (reference) | | | 0.82 (0.74, 0.90) | 0.73 (0.65, 0.81) | 0.91 (0.89, 0.94) | 0.91 (0.89, 0.93) | 0.98 (0.95, 1.00) |
| Osteoarthritis of knee | 1.00 (reference) | | | 0.75 (0.69, 0.81) | 0.47 (0.42, 0.51) | 0.80 (0.78, 0.82) | 0.81 (0.79, 0.82) | 0.91 (0.89, 0.93) |
|  |  | | |  |  |  |  |  |
| 3) excluding participants with a history of cancer at baseline (n = 222,369) | | | | | | | | |
| Osteoarthritis of hip and/or knee | 1.00 (reference) | | | 0.79 (0.74, 0.84) | 0.57 (0.53, 0.61) | 0.84 (0.83, 0.86) | 0.85 (0.83, 0.86) | 0.93 (0.92, 0.95) |
| Osteoarthritis of hip | 1.00 (reference) | | | 0.82 (0.74, 0.91) | 0.72 (0.64, 0.81) | 0.91 (0.88, 0.93) | 0.90 (0.88, 0.93) | 0.97 (0.94, 0.99) |
| Osteoarthritis of knee | 1.00 (reference) | | | 0.78 (0.72, 0.84) | 0.49 (0.45, 0.54) | 0.81 (0.79, 0.82) | 0.81 (0.80, 0.83) | 0.91 (0.90, 0.93) |
|  |  | | |  |  |  |  |  |
| 4) restricting to participants with primary care data (n = 109,353) | | | | | | | | |
| Osteoarthritis of hip and/or knee | 1.00 (reference) | | | 0.79 (0.72, 0.85) | 0.57 (0.51, 0.63) | 0.84 (0.83, 0.86) | 0.85 (0.83, 0.87) | 0.93 (0.91, 0.95) |
| Osteoarthritis of hip | 1.00 (reference) | | | 0.82 (0.72, 0.95) | 0.68 (0.58, 0.80) | 0.90 (0.86, 0.93) | 0.90 (0.87, 0.93) | 0.95 (0.91, 0.98) |
| Osteoarthritis of knee | 1.00 (reference) | | | 0.78 (0.70, 0.86) | 0.51 (0.45, 0.58) | 0.82 (0.79, 0.84) | 0.82 (0.80, 0.84) | 0.92 (0.89, 0.95) |
| 5) further adjusting for baseline history of joint injury (n = 242,278) | | | | | | | | |
| Osteoarthritis of hip and/or knee | 1.00 (reference) | | | 0.77 (0.73, 0.81) | 0.56 (0.52, 0.60) | 0.84 (0.83, 0.85) | 0.84 (0.83, 0.85) | 0.94 (0.92, 0.95) |
| Osteoarthritis of hip | 1.00 (reference) | | | 0.81 (0.74, 0.89) | 0.71 (0.64, 0.79) | 0.91 (0.89, 0.93) | 0.90 (0.88, 0.92) | 0.97 (0.95, 0.99) |
| Osteoarthritis of knee | 1.00 (reference) | | | 0.76 (0.71, 0.81) | 0.48 (0.44, 0.52) | 0.80 (0.79, 0.82) | 0.81 (0.80, 0.82) | 0.91 (0.90, 0.93) |
|  |  | | |  |  |  |  |  |
| 6) further adjusting for baseline history of diabetes and cardiovascular disease (n = 242,278) | | | | | | | | |
| Osteoarthritis of hip and/or knee | 1.00 (reference) | | | 0.78 (0.73, 0.82) | 0.56 (0.53, 0.60) | 0.84 (0.83, 0.85) | 0.85 (0.83, 0.86) | 0.94 (0.92, 0.95) |
| Osteoarthritis of hip | 1.00 (reference) | | | 0.81 (0.73, 0.89) | 0.70 (0.63, 0.79) | 0.91 (0.88, 0.93) | 0.90 (0.88, 0.92) | 0.97 (0.95, 0.99) |
| Osteoarthritis of knee | 1.00 (reference) | | | 0.78 (0.72, 0.83) | 0.49 (0.45, 0.54) | 0.81 (0.79, 0.82) | 0.81 (0.80, 0.83) | 0.92 (0.90, 0.94) |
| ^a^ Abbreviations: LE8, Life's Essential 8; SD, standard deviation. | | | | | | | | |
| ^b^ Multivariable Cox proportional regression was adjusted for age (continuous), sex (male or female), race (White, Asian, Black, or others), Townsend deprivation index (categorical, quartiles), drinking status (current, previous, or never), and education levels (low, moderate, or high). | | | | | | | | |
|  |  |  |  |  |  |  |  |  |
